# Relationship between nurses’ perception of ethical climates and job satisfaction in Jimma University Specialized Hospital, Oromia region, south west Ethiopia

**DOI:** 10.1186/s12912-019-0365-8

**Published:** 2019-08-29

**Authors:** Muktar Abadiga, Gugsa Nemera, Endalew Hailu, Getu Mosisa

**Affiliations:** 1 School of Nursing and midwifery, Institute of Health Sciences, Wollega University, Nekemte, Ethiopia; 2 School of Nursing and Midwifery, Institute of Health Sciences, Jimma University, Jimma, Ethiopia

**Keywords:** Ethical climates, Job satisfaction, Nurses, JUSH

## Abstract

**Background:**

The ethical climate is one aspect of an organization which refers to the shared perceptions of ethically correct behaviors and way of handling ethically deviated behaviors. Increased awareness of the complexity of ethical issues in the health care setting has fueled interest in nursing ethics. However; there is limited information on the relationship between nurses’ perception of ethical climate and job satisfaction globally and no study was done on this issue particularly in Ethiopia. Therefore, this study was aimed to assess the relationship between nurses’ perception of ethical climates and job satisfaction in Jimma University Specialized Hospital, southwest Ethiopia, 2016.

**Methods:**

Institutional based cross-sectional study was conducted on 266 nurses in Jimma University Specialized Hospital from March to April 2016. The study participants were invited by using simple random sampling method. Data were collected using self-administered questionnaires and were entered into Epidata 3.1 and analyzed using SPSS Version 20.0. Pearson’s correlation was used to assess the correlation between each dimension of the hospital ethical climate and job satisfaction of nurses. Variables significant at bivariate analysis (*P* < 0.25) were considered as a candidate for the multivariable linear regression analysis. All analyses were conducted at the 0.05 significance level.

**Results:**

The percentage mean score for ethical climate and job satisfaction were 53.4 and 51.3% respectively. Law and code climate significantly influenced job satisfaction (β = 1.53, *p* = 0.000). Caring climate also significantly influenced nurses job satisfaction (β = 0.99, *p* = 0.000). The result also showed that an independence climate significantly influenced job satisfaction (β = 0.62, *p* = 0.041). On the other hand, rule climate and instrumental climate did not significantly affect job satisfaction (β = 0.380, *p* = 0.409 and β = − 0.208, *p* = 0.290 respectively). The adjusted R square was 0.601, indicating that 60.1% of the variations in job satisfactions was explained by ethical climate variables.

**Conclusion:**

The different dimensions of ethical climates have a negative or positive impact on nurses’ job satisfaction and maintaining a positive ethical climate is key to increasing nurses’ job satisfaction.

## Background

Ethical climate refers to the shared perceptions of ethically correct behaviors and way of handling ethically deviated behaviors [1]. In the health care setting, it is an organizational specific condition that enables the discussion on the clients’ health problems, and provide a framework for ethical decision making [2, 3]. Increased awareness of the complexity of ethical issues in health care, as well as those confronted by nurses, powered interest in nursing ethics [4]. There are five dimensions of Ethical climates: law and codes climate, caring climate, rules climate, independence, and instrumental climates. The most important concern in caring climate is what is best for others and people look out for each other’s good. In law and code climate, employees are expected to respect the law as well as codes of conduct. The concern in rule climate is the degree to which employees strictly adhere to the rules and mandates of the organization. Fulfillment of individual interests is the focus of instrumental climate and it refers to the degree to which employees look out for their self-interest. In independence Climate, employees are expected to be guided by their personal moral beliefs [1]. Job satisfaction, on the other hand, is a complex and multidimensional concept which refers to an internal state of mind of an individual. The difference between what a person gains from his or her job and what he or she expects determines the level of job satisfaction [5, 6].

The increasing turnover of nurses is highly attributed to poor job satisfaction [7]. Job satisfaction is essential for the provision of quality services and dissatisfaction with work can cause poor job performance, lower productivity, and staff turnover and is costly to organizations [8].

According to a survey done in Ethiopia, 50.6% of nurses responded that they were not satisfied with their jobs [7]. Predictors of job satisfaction have mainly focused on demographic variables and work attitudes, but little attention has been given to their relationship with ethics-related issues. However; job satisfaction is influenced by the nature of the ethical climate in the hospital and other health care setting. Employees who perceive their organization to be ethical are likely to perceive their organizations as being fair to them [9].

Different researchers have suggested that the promotion of an ethical climate enables employees to increase their level of job satisfaction. A study done by Morrison indicated that poor workplace relationships will affect the level of job satisfaction. This suggests that individuals who believe that employees are expected to follow the laws and ethical codes of profession and organizations rule are more satisfied with their jobs [10]. In the USA, more than half of the variance in the job satisfaction is explained by the perceived ethical climates which indicates that when the organization’s ethical climate is poor, the employees become dissatisfied with their jobs [11].

In Egypt, almost half of the variation in nurses overall job satisfaction is due to the variation in organizational ethical climates. When the condition of ethical climate within an organization is poor the employees become dissatisfied with their jobs, the service to be delivered is poor in quality and unhealthy competition exists, which also results in turnover intention among employees [12].

However; there is limited information on the relationship between nurses’ perception of ethical climate and job satisfaction globally, and no study was done on this issue particularly in Ethiopia. Therefore, this institutional-based cross-sectional study was conducted to examine nurses’ perception of ethical climate and their relationship with job satisfaction in JUSH.

## Methods and materials

### Study setting and population

This study was conducted at Jimma University Specialized Hospital (JUSH) from March to April 2016. The institutional-based cross-sectional study design was used to assess the relationship between nurses’ perception of ethical climates and job satisfaction in JUSH. The source population was all nurses working in Jimma University Specialized Hospital (JUSH) and all sampled nurses were the study population. All nurses who have been working in the hospital were included in the study and those who had work experience of fewer than 6 months were excluded.

### Sample size determination and sampling techniques

The sample size of the study was calculated using the formula for estimation of a single population proportion. Since the source populations were 515 nurses which were below 10,000, finite population correction was used, and by adding a non-response rate of 20%, a total of 266 nurses were involved in the study. Simple random sampling method was used to invite the study participants.

### Data collection tool and procedures

Questionnaires which have three parts were applied for data collection: a demographic questionnaire, ethical climate questionnaire (ECQ), and job satisfaction scale (JSS). Ethical climate questionnaire (ECQ) of Victor and Cullen was adapted for evaluating the ethical climate [13]. The ethical climate was measured using the 26-item ethical climate questionnaires (ECQ). Six items were measuring caring climate, six items measuring instrumental climate, five items measuring law and code climate, four items measuring rules climate, and five items measuring independence climate. The items were measured on a five-point Likert-type scale 1 = completely false; 5 = completely true). Job satisfaction was measured using the adapted Minnesota job satisfaction questionnaire [14]. There were about 24 items used to measure nurse’s job satisfaction. Responses to all item scales were anchored on a five (5) point Likert scale for each statement which ranges from (1 = completely dissatisfied to 5 = completely satisfied). Data was collected by 3 trained BSc nurses for about 1 month.

### Data processing and analysis

After data collection, each questionnaire was checked visually for completeness. Data were entered into Epi Data Version 3.1 and was analyzed using SPSS version 20.0. The result was analyzed through descriptive statistics such as mean and standard deviation followed by the application of inferential statistics. Mean scores and standard deviations were computed to determine the levels of nurses’ perception of ethical climates and job satisfaction. Pearson’s correlation was used to assess the correlation between each dimension of the hospital ethical climate and job satisfaction of nurses. Variables significant at bivariate analysis (*P* < 0.25) were considered as a candidate for the multivariable linear regression. To further distinguish the contribution of ethical work climate on job satisfaction, coefficient of determination (R2) was used. All analyses were conducted at the 0.05 significance level.

### Data quality control

Since the study units were nurses and assumed to have a common understanding on the tool no need of translating the questionnaires into local languages and the original language prepared in the English version was used to collect the data from the respondents. The training was given to the supervisor to control the quality of the data being collected. A pre-test was done on nurses working in Shenen Gibe hospital by taking 5% of a sample size before the actual data collection and some modification of the questionnaire was made based on pre-test result. The one-day training was given to orient data collectors and supervisors.

## Results

### Socio-demographic characteristics of the respondents

Out of the total 266 sampled nurses in the hospital, 261 of them volunteered to participate and were provided the self-administered questionnaire where only 252 returned the questionnaire making the response rate 94.7%.

Majority of the respondents, 134 (53.2%) were females and the rest were males. Majority of the respondents, 113(44.8%) were Amhara in ethnicity. One hundred thirty-nine (55.2%) were in the age group 25–29 years followed by 30–34 years, 58(23%). One hundred two (40.5%) of the respondents were protestant by religion followed by orthodox, 100 (39.7%). One hundred forty-nine (59.1%) were single and 99 (39.3%) were married. One hundred thirty-six (54.0%) of the respondents were diploma nurses and the highest reported place of assignment of the nurse was medical ward 91 (36.1%). One hundred five (41.7%) answered as their income is less than 2000 birr. Two-hundred four (81%) of the respondents were served 1–5 years followed by 6 months to 1 year 27 (10.7%) (Table 1).

**Table 1:** Sociodemographic characteristics of nurses, Jimma University Specialized Hospital, March 2016, (*n* = 252)

Sociodemographic characteristics	Frequency (*N* = 252)	Percent (%)
Gender		
Female	134	53.2
Male	118	46.8
Total	252	100
Ethnicity		
Oromo	106	42.1
Amhara	113	44.8
Gurage	9	3.6
Tigre	7	2.8
Others (yem, wolayita, kefa)	17	6.7
Total	252	100
Age		
20–24 years	48	19.0
25–29 years	139	55.2
30–34 years	58	23
35–39 years	6	2.4
40–44 years	1	0.4
Total	252	100
Religion		
Muslim	42	16.7
Orthodox	100	39.7
Protestant	102	40.5
Catholic	5	2
Wakefata	3	1.2
Total	252	100
Marital status		
Married	99	39.3
Single	149	59.1
Widowed	3	1.2
Divorced	1	0.4
Total	252	100
Educational status		
Diploma	136	54.0
Degree	116	46.0
Total	252	100
Working unit		
Medical	91	36.1
Surgical	60	23.8
Maternity	49	19.4
Pediatrics	24	9.5
Psychiatry	10	4.0
Others (OPD, OR,ICU)	18	7.1
Total	252	100
Salary		
< 2000 Ethiopian birr	105	41.7
2000–3000 Ethiopian birr	45	17.9
3000–4000 Ethiopian birr	93	36.9
> 4000 Ethiopian birr	9	3.6
Total	252	100
Work experience:		
6 month- 1 year	27	10.7
1–5 years	204	81.0
6–10 years	21	8.3
Total	252	100

### Level of ethical climates and job satisfaction

Descriptive statistics were performed to find out means and standard deviations of Ethical Climates and Job Satisfaction. Table 2 reports descriptive statistics including means and standard deviation for samples. The different types of EC (law and code, rules, caring, independence and instrumental,) were examined. The mean score of caring climate dimension is 13.78 ± 6.41 SD with a range of 6 to 28.

**Table 2:** Means and standard deviations of ethical climates and job satisfaction among nurses working in JUSH, March 2016, (*n* = 252)

Variables	Mean	SD	Minimum	Maximum
Instrumental climates	21.03	5.94	6	30
Independence climates	12.68	4.501	5	25
Rule climates	10.21	4.36	4	20
Law and code climates	11.78	5.04	5	25
Caring climates	13.78	6.41	6	28
Total ethical climate measurement	69.47	1.71	37	119
Total job satisfaction scales	61.60	2.41	26	115

The mean score for law and code climate dimension is 11.78 ± 5.04 SD with a range of 5 to 25. The mean score for the rule climate dimension is 10.21 ± 4.36 SD with a range of 4 to 20. The mean score of independence climate dimension is 12.68 ± 4.501 SD with a range of 5 to 25. The mean score for instrumental climate dimension is 21.03 ± 5.94 SD with a range of 6 to30. The overall mean score for the ethical climate was 69.47 ± 1.71 with a range of 37to 119 and that of job satisfaction was 61.6 ± 2.41with a range of 26 to115. The percentage mean score for law and code climate was 47.12%, for rule climate 51.05%, for caring climate 45.93%, for independence climate 50.72% and instrumental climate 70.1%. The percentage mean score for the overall ethical climate was 53.4% and the percentage mean score for overall job satisfaction was 51.3%. Percentile scores of 25 or lower indicate low, percentile scores between 26 and 74 displays moderate and percentile scores of 75 or higher represent the high level. Therefore, the percentage mean score obtained in this study indicates a moderate level of ethical climate and job satisfaction were observed among nurses at JUSH. The result showed moderate levels of hospital ethical climate and moderate levels of job satisfaction among nurses working in JUSH (Table 2).

**Fig. 1:**
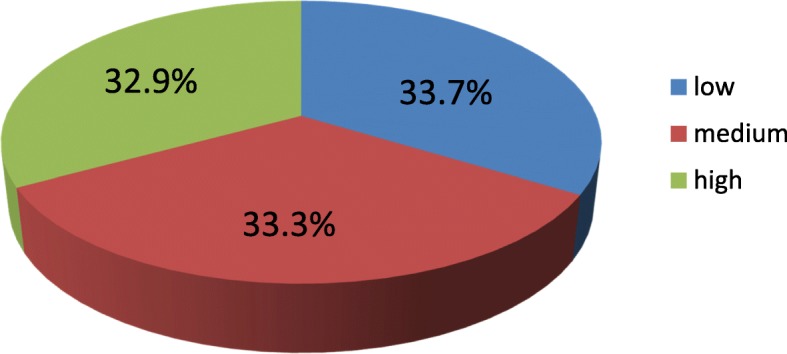
Tertile classification indicating the level of ethical climates among nurses in JUSH, March 2016

**Fig. 2:**
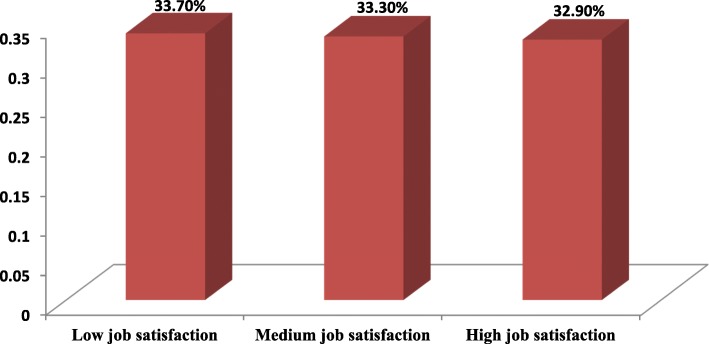
Tertile classification indicating level of job satisfaction among nurses in JUSH, March 2016

The tertile classification was also used to determine the level of ethical climate and job satisfaction. The following figure shows the tertile classification of ethical climate and job satisfaction among nurses in JUSH (Figs. 1 and 2).

### Linear regression assumption test

Linear regression assumptions of normal distribution were checked by Kolmogorov-Smirnov and Shapiro-Wilk test, as well as skewness and kurtosis. The test result showed that the variables were normally distributed. Therefore, data transformation was not needed and the original data were used for subsequent analysis.

### Collinearity diagnosis

Multi-collinearity or correlations between each dimension of ethical climates were checked using VIF and tolerance test. The values of tolerance for all dimension of ethical climate were greater than 0.2. Similarly, all values of VIF were less than 10. Therefore, there was no issue of collinearity when overall job satisfaction was taken as the dependent variable.

### Bivariate linear regression analysis

#### T-test

T-test result showed that there were no significant differences in nurses job satisfaction levels between males and females (t = − 2.99, *p* > 0.05), diploma’s and bachelor’s nurses (t = − 0.05, *p* > 0.05).However; there were a significant differences in nurses job satisfaction levels between staff nurse and manager nurse (t = − 1.49, *P* < 0.05).

#### ANOVA

One-way ANOVA was also used to examine the differences in nurses’ job satisfaction levels. There were significant differences in the means of nurses’ job satisfaction levels between age groups (*F* = 3.967, *P* < 0.05), monthly salary (*F* = 12.71, *P* < 0.05) and working unit (*F* = 2.33, *P* < 0.05), but no significant differences in the means of nurses’ job satisfaction levels between year of experiences (*F* = 1.08, *P* > 0.05) and marital status (*F* = 1.406, *P* > 0.05).

Bivariate linear regression analysis was done to investigate how much each sociodemographic variable predicts job satisfaction. Many of sociodemographic variables were significantly affected job satisfaction at the bivariate level and these variables include ethnicity (Oromo, Amhara), age (20–24 years, 30–34 years), working unit (surgical ward and maternity ward) and salary (< 2000 birr) (Table 3).

**Table 3:** Bivariate linear regression analysis result of sociodemographic characteristics among nurses in JUSH, March 2016, (*n* = 252)

Sociodemographic Variables	Unstandardized β coefficient	SE	t value	*P* value	95% confidence interval	
					Lower bound	Upper bound
Ethnicity						
Oromo	8.96	3.02	2.96	0.003	3.01	14.91
Amhara	−9.46	2.99	−3.16	0.002	−15.35	−3.56
Gurage	−9.72	8.15	−1.19	0.234	−25.79	6.34
Tigre	2.17	9.23	0.23	0.814	− 16.01	20.37
Age in years						
20–24 years	8.62	3.82	2.25	0.025	1.08	16.16
25–29 years	2.89	3.04	0.95	0.342	−3.10	8.90
30–34 years	−12.24	3.52	− 3.47	0.001	− 19.18	−5.30
35–39 years	1.60	9.95	0.16	0.872	−18.00	21.21
Religion						
Muslim	−0.17	4.07	−0.04	0.966	−8.20	7.84
Orthodox	4.51	3.09	1.46	0.146	−1.57	10.59
Protestant	−3.11	3.08	−1.00	0.314	−9.19	2.96
Catholic	−11.63	10.86	−1.07	0.285	−33.02	9.76
Marital status						
Married	−2.03	3.10	−0.65	0.513	−8.15	4.08
Single	2.80	3.08	0.90	0.364	−3.27	8.87
Widowed	−23.54	13.92	−1.69	0.092	−50.96	3.86
Diploma	−1.52	3.04	−0.50	0.617	−7.52	4.47
Working unit						
Medical	1.57	3.15	0.49	0.619	−4.64	7.79
Surgical	9.80	3.51	2.79	0.006	2.88	16.71
Maternity	−8.34	3.80	−2.19	0.029	−15.82	−0.86
Pediatrics	−4.07	5.16	−0.78	0.432	−14.24	6.10
Psychiatry	−2.39	7.77	−0.30	0.758	−17.70	12.92
Salary						
< 2000 birr	−9.06	3.02	−2.99	0.003	−15.01	−3.10
2000–3000 birr	21.88	3.71	5.89	0.000	14.57	29.20
3001–4000 birr	−5.67	3.12	−1.81	0.071	−11.82	0.48
Experience						
< 1 year	1.52	4.90	0.31	0.756	−8.13	11.19
1–5 year	3.03	3.86	0.785	0.433	−4.57	10.63
6–10 year	−8.03	5.47	−1.462	0.143	−18.80	2.74

Predictor variables: Sociodemographic variables

Dependent variables: job satisfaction

Pearson’s correlation coefficient was used to determine the correlation between hospital ethical climates and job satisfaction of nurses. Results indicated a significant correlation between hospital ethical climate and nurses’ job satisfaction.

The strongest correlation was observed between law and code climate and job satisfaction (*r* = 0.734, *P* < 0.01). The second strongest correlation exists between caring climate and job satisfaction (*r* = 0.733, *P* < 0.01). Respondents who believed that their hospitals had a caring climate were more satisfied with their job. The third strongest correlation exists between rules climate and job satisfaction (*r* = 0.698, *P* < 0.01).

Also, the study found that the correlation between independence climate and job satisfaction was moderate but significant (*r* = 0.574, *P* < 0.01).

Finally, there was a moderate negative correlation between instrumental climate and job satisfaction and therefore the study suggests that an instrumental climate significantly and negatively influenced job satisfaction (*r* = − 0.313, *P* < 0.01).

Based on these results, the correlation between each dimension of ethical climates and job satisfaction was significant at the level of *P* < 0.01. In this study, the relationship between the overall ethical climate types and job satisfaction was positive, strong and significant (*r* = 0.712, *P* < 0.01). From this, it can be concluded that there was a strong positive and significant relationship between the overall ethical climate dimension and job satisfaction.

Bivariate linear regression analysis was also used to investigate how much each dimension of ethical climate predicts job satisfaction. At the bivariate level, all ethical climate dimensions were significantly associated with nurses’ job satisfaction. The table below shows bivariate linear regression between each dimension of ethical climate and job satisfaction. The result showed that law and code climate positively and significantly affected nurses job satisfaction (β = 3.50, *P* < 0.001). Caring climate significantly and positively influenced nurses job satisfaction (β = 2.75, *p* < 0.001). Independence climate also significantly and positively influenced job satisfaction (β = 3.06, *p* < 0.001). Rule climate positively and significantly affect job satisfaction (β = 3.85, *p* < 0.001). On the other hand, the result also showed that the instrumental climate significantly and negatively influenced job satisfaction (β = − 1.26, *P* < 0.001) (Table 4).

**Table 4:** Bivariate linear regression analysis of the five ethical climate dimensions and job satisfaction among nurses in JUSH, March 2016, (*n* = 252)

Ethical climate Variables	Unstandardized β coefficient	SE	t value	*P* value	95% confidence interval	
					Lower bound	Upper bound
Law and code climate	3.50	0.20	17.07	0.000	3.09	3.90
Rule climate	3.85	0.25	15.42	0.000	3.36	4.34
Caring climate	2.75	0.16	17.05	0.000	2.43	3.07
Independence climate	3.06	0.27	11.07	0.000	2.51	3.60
Instrumental climate	−1.26	0.24	−5.21	0.000	−1.74	−0.79

Predictor variables: ethical climate variables

Dependent variables: job satisfaction

### Multivariable linear regression analysis

Multivariable linear regression was finally conducted to examine the best combination of factors for predicting job satisfaction.

All predictors of job satisfaction with *P* value < 0.25 at bivariate analysis were entered into the multivariable linear regressions model and backward elimination was used to extract factors that best predicts job satisfaction. Many sociodemographic variables that demonstrated a significant bivariate relationship with job satisfaction were not significant in the multivariable regression model. From sociodemographic variables, only benefit/salary and working unit were significantly predicted job satisfaction. From ethical climate dimensions, law and code climate, caring climate and independence climate were significantly predicted job satisfaction.

However; rule climate and instrumental climate were not significantly predicted job satisfaction. Since the aim of this study was to examine the relationship between different ethical climate dimension and job satisfaction, other factors were adjusted for or controlled.

In controlling for other variables, the relationship between ethical climate and job satisfaction decreased (β value decreased) indicating that variables other than ethical climate variables partially mediate the relationship between ethical climate and job satisfaction. To identify the unique contribution of ethical climate, we looked at what ethical climate uniquely contributes when controlling for all the other variables. By controlling the effects of the socio-demographic data, the researcher then determined how well the five subscales of the ethical climate predicted nurses’ job satisfaction levels.

Multivariable linear regression analyses show that law and code climate significantly and positively influenced job satisfaction (β = 1.53, *p* = 0.000). This can be interpreted as a one-unit increase in law and code climate results in an average of 1.53 unit increases in job satisfaction. Caring climate significantly and positively influenced nurses job satisfaction (β = 0.99, *p* = 0.000).

The interpretation is a one-unit increase in a caring climate leads to an average of 0.99 unit increase in job satisfaction. The result also showed that an independence climate significantly and positively influenced job satisfaction (β = 0.62, *p* = 0.041). It means that a one-unit increase in independence climate results in an average of 0.62 unit increases in nurses’ job satisfaction.

On the other hand, the result showed that rule climate did not significantly affect job satisfaction (β = 0.380, *p* = 0.409). Therefore, the study couldn’t find out a significant relationship between the rule climate and job satisfaction at the multivariable level.

This study also revealed that an instrumental climate did not significantly influenced job satisfaction (β = − 0.208, *p* = 0.290). This result can be interpreted as a one-unit increase in instrumental climate results in an average of 0.208 unit decrement in job satisfaction of nurses, but not statistically significant.

Therefore, from the five dimensions of ethical climates, law and code climate, caring climates and independence climates significantly contributed to the prediction of job satisfaction. But rule climate and instrumental climate did not contribute much to the prediction. From sociodemographic variables, monthly income and working unit were positively and significantly affected nurses’ job satisfaction in the final model.

In the multivariable regression, the model is significant (*F* = 76.46, *P*-value = 0.000) and has an adjusted *R2* = 0.601. The adjusted R2 of 0.601 demonstrates the actual percentage of the variable which explains the entire model, that is, 60.1%. The adjusted R2 of 60.1% of this model indicates that ethical climate variables can explain only 60.1% of the variance or change in job satisfaction. Hence, 48.9% of the variations in job satisfaction were explained by other factors (Table 5).

**Table 5:** Summary of multivariable linear regression for job satisfaction among nurses’ working in JUSH, March 2016, (*n* = 252)

Variables	Unstandardized β coefficient	SE	t value	*P* value	95% confidence interval	
					Lower bound	Upper bound
Law and code climate	1.53	0.36	4.24	0.000	0.817	2.23
Caring climate	0.99	0.33	3.04	0.003	0.35	1.64
Independence climate	0.62	0.30	2.03	0.043	0.02	1.22
Rule climate	0.38	0.46	0.82	0.409	−0.52	1.28
Instrumental climate	−0.208	0.19	−1.06	0.290	−0.59	0.17

Adjusted R square = 0.601(60.1%), *F* = 76.46, *P* = .000

Dependent variable: job satisfaction

The model equation based on the information obtained was as follows:

Job satisfaction = 17.73 + 1.53 (Law and code climate) + 0.99 (Caring climate) + 0.62 (Independence climate) + 5.39.

## Discussion

This study aimed to determine the relationship between nurses’ perception of ethical climate and job satisfaction in JUSH. The findings of this study indicated that the ethical climate variables were strongly correlated with job satisfaction. It refers to the fact that poor ethical climate in the hospital worse the level of nurses’ job satisfaction. After adjustment for confounding factors, it was observed that nurses’ job satisfaction was significantly affected by all dimensions of ethical climate except rule and instrumental climates.

The percentage mean score for the ethical climate was 53.4% and the percentage mean score for job satisfaction was 51.3%. These indicate that the level of ethical climate and job satisfaction of nurses are moderate. This finding is low relative to the study done in Egypt in which the percentage mean score for ethical climate and job satisfaction were 64.2 and 57.2% respectively [12]. The possible reason for this discrepancy is that in JUSH there is poor adherence to legal and professional codes of ethics and most nurses concerned with what is best for themselves and are mostly out for themselves which leads to a lower level of ethical climate and job satisfaction. The level of job satisfaction in this current study (51.3%) is somewhat higher than the study done among health professionals working in Jimma University Specialized Hospital in which the level of job satisfaction is only 41.4% [15].

The results of this study revealed that there is a positive and significant correlation between caring climate and job satisfaction (β = 0.99, *p*-value = 0.003). This means respondents who believed that their hospitals had a caring climate were more satisfied with their jobs. This finding is consistent with research results of the study conducted in Taiwan and Teaching hospital of the Kerman University of Medical Science, and their results indicated that there was a significant positive relationship between caring ethical climate and job satisfaction [16, 17]. However; the result of this study contradicts the finding of the study done in Singapore [18] which couldn’t find a significant relationship between caring ethical climate and job satisfaction.

The result of this study also showed that there was a positive and significant correlation between law and code climate and job satisfaction (β = 0.335, *p*-value = 0.000). It could be said that climates encouraging law and code climate results in eliminating ambiguity during controlling ethical situations; and consequently, results in increased satisfaction of staff. This finding is in line with the findings of a study conducted in Iran which concluded that law and professional codes climate type was significantly and positively correlated with job satisfaction [19]. But this result doesn’t go in line with the study conducted in Taiwan in which Law and code climate type did not significantly affect job satisfaction and therefore, it couldn’t find out a relationship between the laws and codes ethical climate and job satisfaction [16].

Similarly, this study found a significant positive correlation between independence climate and job satisfaction (β = 0.62, *p*-value = 0.043). This result indicates that if nurses act according to their own personal and moral beliefs, their satisfaction of the job will be increased or if they are expected to follow their own personal and moral beliefs, they will be more satisfied with their jobs. This finding is consistent with the study conducted in Tehran which pointed out that an independent climate significantly and positively influenced the overall job satisfaction which supports the result of this current study [20].

On the contrary, the findings of this study showed that the rules climate did not significantly affect nurses job satisfaction (β = 0.380, *p*-value = 0.409). This finding is consistent with the research results of a study conducted in the Southeastern United States [11]. This finding is in contrary to the findings of the study done in Tehran, that shows as organizational rules and procedures have a significant impact on work satisfaction [20].

Finally, among the dimensions of the ethical climate, instrumental climate also had no significant impact on job satisfaction (β = − 0.208, *p*-value = 0.290). This finding is consistent with previous research results of the study done in Iran which didn’t find any significant relationships between instrumental climate and job satisfaction [19]. However; this result contradicts the research results of the study done in Turkey, which found a significant and negative correlation between instrumental climate and job satisfaction [21].

The overall correlation between ethical climate and job satisfaction has an adjusted R square of 0.601, demonstrating that the five independent variables of care, law and code, rules, instrument and independence combined explained 60.1% of the total factors of Job Satisfaction. That is, 60.1% of the variation in nurses overall job satisfaction can be explained by the variation in the ethics variables in the hospital. This research finding is nearly consistent with the study done in the Southeastern United States with adjusted R- square of 0.718 and in Nigeria with adjusted R- square of 0.58 [22, 23]. However; this finding is relatively high to the results of studies done in Singapore (adjusted *R*-square = 0.3516) and in China (adjusted R square = 0.338) [18, 24]. The possible reason for this discrepancy is that there are poor utilization and non-adherence to ethics and ethics-related elements in our hospital setting, and on the other hand in the hospital setting of other countries there might be strict adherence to code of ethics and proper utilization of these ethics which enhances employee job satisfaction.

### Limitation of the study

Causality cannot be confirmed since the research design was cross-sectional. Thus, in this study, the relationship between types of ethical climate and job satisfaction cannot be considered as cause and effect. Absence of literature on this topic forces the researchers to compare and discuss the finding with the study done in other countries. Since this is the first study on this topic in Ethiopia, the findings of this study have been compared and discussed with the international evidence.

## Conclusion

The findings of this study revealed that the level of nurses’ perception of ethical climates and job satisfaction was moderate. Some dimensions of ethical climates existing in the hospital have a significant impact on nurses’ job satisfaction whereas other dimensions have no significant impact on nurses’ job satisfaction. A caring climate, law and code climate and an independence climate were positively and significantly affected nurses’ job satisfaction whereas instrumental climate and rule climate had no significant impact on nurses’ job satisfaction.

## Data Availability

The data used during the current study are available from the corresponding author on request.

## Abbreviations

EC: Ethical climates
ECQ: Ethical climate questionnaires
IRB: Institutional Review Board
JS: Job Satisfaction
JSS: Job Satisfaction Scales
JU: Jimma University
JUSH: Jimma University Specialized Hospital
PI: Principal Investigators
SPSS: Statistical Package for Social Science
